# Lysosomal and phagocytic activity is increased in astrocytes during disease progression in the SOD1 ^G93A^ mouse model of amyotrophic lateral sclerosis

**DOI:** 10.3389/fncel.2015.00410

**Published:** 2015-10-15

**Authors:** David J. Baker, Daniel J. Blackburn, Marcus Keatinge, Dilraj Sokhi, Paulius Viskaitis, Paul R. Heath, Laura Ferraiuolo, Janine Kirby, Pamela J. Shaw

**Affiliations:** Department of Neuroscience, Sheffield Institute for Translational Neuroscience, University of SheffieldSheffield, UK

**Keywords:** microarray, superoxide dismutase 1, cholesterol/steroid, neurodegeneration, motor neuron

## Abstract

Astrocytes are key players in the progression of amyotrophic lateral sclerosis (ALS). Previously, gene expression profiling of astrocytes from the pre-symptomatic stage of the SOD1^G93A^ model of ALS has revealed reduced lactate metabolism and altered trophic support. Here, we have performed microarray analysis of symptomatic and late-stage disease astrocytes isolated by laser capture microdissection (LCM) from the lumbar spinal cord of the SOD1^G93A^ mouse to complete the picture of astrocyte behavior throughout the disease course. Astrocytes at symptomatic and late-stage disease show a distinct up-regulation of transcripts defining a reactive phenotype, such as those involved in the lysosome and phagocytic pathways. Functional analysis of hexosaminidase B enzyme activity in the spinal cord and of astrocyte phagocytic ability has demonstrated a significant increase in lysosomal enzyme activity and phagocytic activity in SOD1^G93A^ vs. littermate controls, validating the findings of the microarray study. In addition to the increased reactivity seen at both stages, astrocytes from late-stage disease showed decreased expression of many transcripts involved in cholesterol homeostasis. Staining for the master regulator of cholesterol synthesis, SREBP2, has revealed an increased localization to the cytoplasm of astrocytes and motor neurons in late-stage SOD1^G93A^ spinal cord, indicating that down-regulation of transcripts may be due to an excess of cholesterol in the CNS during late-stage disease possibly due to phagocytosis of neuronal debris. Our data reveal that SOD1^G93A^ astrocytes are characterized more by a loss of supportive function than a toxic phenotype during ALS disease progression and future studies should focus upon restorative therapies.

## Introduction

Amyotrophic lateral sclerosis (ALS) is a neurodegenerative disorder characterized by progressive loss of motor neurons and resulting muscular weakness leading to death on average within 2–3 years of symptom onset. ALS occurs in approximately 2 per 100,000 population with incidence enhanced by: increasing age; gender (1.6:1 male to female ratio); and genetic susceptibility. Five to 10% of cases of ALS are familial (fALS) and multiple causative genes have been identified (Goodall et al., 2012). The most common known genetic cause of ALS is a mutation in the *C9ORF72* gene (Dejesus-Hernandez et al., 2011; Renton et al., 2011), implicated in approximately 7% of sporadic cases (sALS) and 43% of fALS (Cooper-Knock et al., 2012). Mutations in *SOD1* (Cu/Zn superoxide dismutase (1) are the second most common genetic cause of ALS, and account for 10–20% of fALS (Cudkowicz et al., 1997). Transgenic mice expressing mutant human SOD1 (mSOD1) are widely used as an animal model of ALS, allowing for detailed study of motor neurons and the surrounding glia during disease progression (Gurney et al., 1994; Turner and Talbot, 2008). Using the SOD1^G93A^ model of ALS originally developed by Gurney et al. (1994) we have developed the model on a homogeneous background featuring highly consistent disease progression (Mead et al., 2011), which allows analysis of discrete time-points in disease.

Astrocytes play important roles in development, blood flow, homeostasis, synaptic function, metabolism, and formation of the blood brain barrier (Sofroniew and Vinters, 2010). In ALS, astrocytes become activated in a process called reactive astrogliosis, in which astrocytes become hypertrophic and release increased levels of chemokines and cytokines (Blackburn et al., 2009; Philips and Robberecht, 2011). Astrocytic protein inclusions containing mSOD1 are an early feature of disease in the mSOD1 mouse model (Bruijn et al., 1997). Selective expression of mSOD1 in astrocytes alone failed to provoke an ALS phenotype (Gong et al., 2000), but silencing mSOD1 expression in astrocytes significantly slowed disease progression in the SOD1^G37R^ mouse model (Yamanaka et al., 2008), without affecting the level of astrogliosis. Studies using chimeric mice have shown normal motor neurons develop features of ALS pathology when surrounded by mSOD1-expressing glial cells (Clement et al., 2003). *In vitro* mSOD1 astrocytes are selectively toxic to motor neurons (Nagai et al., 2007; Bilsland et al., 2008; Cassina et al., 2008; Díaz-Amarilla et al., 2011) and this toxicity has been replicated in astrocytes derived from human cases of sporadic and familial MND (Haidet-Phillips et al., 2011; Re et al., 2014). In concert with these increased toxic properties of astrocytes, ALS is characterized by a loss of essential supportive behavior in these cells. Glutamate re-uptake, a process in which astrocytes play a major part, is compromised in the ALS spinal cord, motor cortex, and somatosensory cortex (Rothstein et al., 1995). *In vitro*, the presence of mutant SOD1 within astrocytes leads to altered GluR2 AMPA receptor subunit expression in co-cultured neurons, increasing their susceptibility to excitotoxic damage (Van Damme et al., 2007). Mutant SOD1 is also suggested to render astrocytes vulnerable to excitotoxic damage through activation of mGluR5 (Rossi et al., 2008) which can be rescued by the anti-apoptotic Bcl-X_L_ leading to improved motor performance (Martorana et al., 2011).

Gene expression profiling using microarray technology has been used to delineate specific pathways that may serve as therapeutic targets in ALS. Laser capture microdissected (LCM) motor neurons from the SOD1^G93A^ mouse model at pre-symptomatic (~60 day), symptomatic (~90 day), and late-stage (~120 day) have been used previously to show the change in motor neuron transcriptional behavior throughout the disease course (Ferraiuolo et al., 2007). Microarray analysis of LCM astrocytes, taken from the pre-symptomatic time-point revealed dysregulated metabolic homeostasis, in particular a reduction in lactate provision from astrocytes to motor neurons (Ferraiuolo et al., 2011a). Due to the complexity of the disease process, a transcriptomic approach is needed so that interactions between the multiple disease mechanisms can be identified (Ferraiuolo et al., 2011b). This approach can then be used to study the specific changes that occur in astrocytes during ALS and how these affect their interaction with motor neurons. The aim of this study was to perform microarray analysis of laser-captured SOD1^G93A^ astrocytes from the symptomatic and late-stage time-points to complement the previous work performed by Ferraiuolo et al. (2011a) and to reveal the full extent of astrocytic behavior during disease progression. The data show that an activated phenotype becomes apparent at the symptomatic stage which follows through to late-stage and is characterized by an up-regulation of transcripts involved in lysosomal and phagocytic pathways. We hypothesized that these pathways are up-regulated at the functional level. To test this, we performed enzymatic assays and *in vitro* experiments using fluorescently labeled NSC34 cell debris to confirm an intrinsic lysosomal and phagocytic up-regulation in SOD1^G93A^ astrocytes. At the late-stage, a dysregulation in transcripts involved in many steps of cholesterol processing also occurs and immunohistochemistry has confirmed an altered distribution of cholesterol processing enzymes in late-stage lumbar spinal cord. In combination with the pre-symptomatic data of Ferraiuolo et al. (2011a), these findings provide a detailed map of astrocyte behavior throughout disease and point to a loss of supportive function as the overwhelming astrocyte phenotype that emerges during the disease course.

## Materials and methods

### The SOD1^G93A^ mouse model

The SOD1^G93A^ mouse model, staining, LCM, and microarray analysis were performed as in Ferraiuolo et al. (2011a). Mice used were male SOD1^G93A^ transgenic mice B6SJL-Tg (SOD1^G93A^) 1 Gur/J, which had been backcrossed onto C57Bl/6 J Ola/Hsd (Harlan) for over 20 generations (Mead et al., 2011), and their non-transgenic (NTg) littermates were used. Three mice were used for disease and control groups at the symptomatic (90 days) and late-stage (120 days) time-points. All procedures were performed according to UK Home Office regulations and in accordance with guidelines specified by The University of Sheffield Ethics Committee.

### Rapid immunohistochemistry (IHC) using ALDH1L1

Rapid IHC staining of astrocytes, to allow rapid isolation of cells and preservation of RNA quality, was performed as previously described (Ferraiuolo et al., 2011a). Tissue sections (10 μm) were stained with primary antibody (rabbit polyclonal anti-ALDH1L1, Abcam, #ab 79727) at 1:50 dilution and secondary antibody and ABC reagent were from the Vectastain Rabbit IgG kit (Vector Labs # PK-6101).

### Laser capture of astrocytes

A minimum of 1500 astrocytes per animal were captured by LCM using the Arcturus Pixcell II microdissection instrument (Applied Biosystems, Carlsbad, CA) using Arcturus Capsure Macro LCM Caps (Applied Biosystems #LCM0211). RNA was extracted from captured cells using the Arcturus Picopure RNA Isolation kit (Applied Biosystems #12204-01) and tested for RNA quantity using the Agilent Nanodrop spectrophotometer ND-1000 (Agilent Technologies, Santa Clara, CA) and for quality using the RNA 6000 PicoChip kit (Agilent # 5067-1513).

### Microarray data analysis and gene ontology categorization

RNA was linearly amplified using the Two Rounds Amplification kit (Affymetrix) as per manufacturer's instructions. cRNA was generated using the GeneChip Expression 3′ Amplification Reagents for IVT labeling (Affymetrix). Fifteen micrograms of cRNA for each of 3 × SOD1^G93A^ and 3 × NTg mice was fragmented (Genechip reagents, Affymetrix) and 12.5 μg hybridized onto Mouse Genome 430 2.0 Genechips (Affymetrix). Prior to analysis probe sets were removed from the analysis if all samples were called absent using the MAS5 algorithm within Bioconductor (Gentleman et al., 2004). Initial GeneChip analysis for determination of differentially expressed transcripts was then performed using Genespring GX (Agilent) with the Probe Logarithmic Intensity Error (PLIER) algorithm. For Gene Ontology categorization, genes with *p* ≤ 0.05 and fold-change ≥2 were analyzed using the Database for Annotation and Visualization (DAVID) in combination with NetAffx Analysis Centre (www.affymetrix.com) and Genecards V3 (www.genecards.org).

### qPCR validation

The amplified samples used in the microarray analysis were diluted to 12.5 ng/μl and used for qPCR validation. Only selected genes were validated via q-PCR due to the limited availability of RNA from late stage animals following microarray analysis. As a result some pathways were only validated by functional tests. Genes were chosen based upon their presence in key pathways of interest. Optimization of primer concentrations was performed as described previously (Ferraiuolo et al., 2007). qPCR was performed in 10 μL reaction volumes using 5 μL Brilliant III Ultra Fast SYBR® Green qPCR mastermix (Agilent). Primer sequences, concentrations and the PCR programme used are shown in Supplementary Table 1. To calculate relative expression differences between SOD1^G93A^ astrocytes and NTg controls the 2[-Delta Delta C(T)] method was used (Livak and Schmittgen, 2001). *Gapdh* was chosen as the housekeeping gene due to a consistent expression level across all GeneChips and based upon previous optimization at the presymptomatic time-point (Ferraiuolo et al., 2011a).

### Astrocyte cell culture

Primary astrocyte cultures were set up as previously described (Ferraiuolo et al., 2011a). Cortices from 0 to 2 day old SOD1^G93A^ mice and their NTg littermates were used to make separate cultures of astrocytes. The presence of the transgene for each culture was ascertained using PCR for each pup tail clip. Cells were cultured in DMEM containing 10% FBS until 100% confluence was reached, at which time flasks were shaken overnight at 225 rpm at 37°C followed by mild trypsinisation (Saura et al., 2003) to separate astrocytes from microglia. Astrocytes were defined as ≥95% purity before use in experiments using a FACSCalibur flow cytometer (BD) and antibodies against GLAST (Miltenyi Biotech) and CD11b (Ebioscience) to check for microglial presence.

### β hexosaminidase activity assay

Symptomatic and late-stage mice were euthanized via intraperitoneal injection of pentobarbital and perfused with PBS. Spinal cords were dissected, cut just above the lumbar enlargement to give the “upper cord” and “lower cord,” and rapidly frozen in liquid nitrogen. Sections of tissue (~5 mg) were dissected from the thoracic section of the upper cord and the lumbar section of the lower cord on dry ice and then homogenized using a glass tissue homogeniser (Jencons, England). Protein concentration of homogenates was measured using BCA assay (Sigma #B9643) and samples diluted to 1 mg/mL using distilled water. Five microliters of diluted homogenate was added to 500 μL McIlvaine citrate–phosphate pH 4.5 buffer (MV4.5) and 100 μL aliquots taken. All tubes and substrate solution [4-Methylumbelliferyl N-acetyl-β-D-glucosaminide (Sigma) at 1.1 mg/mL in MV4.5] were warmed for 2 min at 37°C, before the substrate was added at timed intervals in 100 μL volumes to all tubes. All tubes were then incubated at 37°C for 10 min and then stopping solution (0.25 M glycine buffer pH10.4) added at timed intervals. Sample fluorescence was then read using a FLUOstar Omega platereader (BMG Labtech) with excitation settings of 365 nm and emission of 450 nm.

### Astrocyte phagocytosis assay

Cell debris was created by placing complete medium containing 1:200 Vybrant Dil (Life Technologies) onto the murine NSC34 motor neuronal cell line for 20 min at 37°C. Cells were washed for 10 min in fresh complete medium and were then placed in serum-free DMEM for 48 h to induce cell death. Cells were collected and counted and stored at −80°C. Neonatal astrocytes from SOD1^G93A^ and NTg mice were seeded at a density of 4 × 10^3^ per well of a 384 well optical plate (Greiner Bio One) and changed to serum-free medium (DMEM/F12 containing 1 × N2) 24 h prior to exposure to cell debris. NSC34 debris was placed onto astrocytes at a concentration of 125 cells/μL in serum-free medium. Following a 48 h incubation at 37°C, cultures were washed 3 times with PBS and fixed with 4% PFA for 15 min at room temperature. As a negative control, cells were treated with decreasing concentrations of Latrunculin A (10, 5, and 1 μM), a commonly used phagocytosis inhibitor that forms a complex with actin filaments and inhibits actin polymerization and elongation. One micromolar was the concentration that less affected astrocyte viability and morphology and was, therefore, used as a negative control for the phagocytosis assay. Cells were treated overnight with 1 μM Latrunculin A (Sigma), the day after cells were washed and cell debris were applied onto the cells as above. Astrocytes were stained with anti-GFAP antibody (Abcam #ab7260) and nuclei were visualized using Hoescht staining. The InCell 2000 (GE) was used for image capture of 9 fields per well (5 wells per sample) and subsequent analysis was performed using InCell Developer Toolbox v.1.9 (GE) with an analysis protocol that counted debris if ≥65% of debris-signal overlapped with GFAP-signal. Confocal images of phagocytosis were taken on a Leica SP5 confocal microscope using a x63/1.4 oil immersion objective lens. Z-stack images were taken at a resolution of 512 × 512 pixels at intervals of 0.12 μm throughout the depth of the cell of interest.

### Immunohistochemistry for SREBP2

Sections of frozen spinal cord (10 μm) were taken from three symptomatic SOD1^G93A^ mice and 3 age-matched NTg controls. Staining was performed as per the manufacturer's protocol using the Vectastain Rabbit IgG kit as used for Aldh1l1 staining. The primary antibody used was Ab282482 (AbCam) at 1:400 dilution in blocking solution. SREBP2 staining was visualized using diaminobenzidine and nuclei were visualized using haemotoxylin. Microscopy was performed using a Nikon Eclipse Ni microscope with a Nikon DS-Ri1 camera using brightfield settings of 9.5 ms exposure for 20X magnified images and 44 ms exposure for 40X magnified images.

### Cholesterol assay

Primary cortical astrocytes isolated from neonate SOD1^G93A^ mice and their NTg littermates were plated in a 96-well plate at a density of 3 × 10^4^ cells/well. Twenty-four hours post-plating cells were either treated with U-18666A 1.25 μM, a cholesterol transport inhibitor, or medium was replaced. Forty-eight hours later cells were fixed and cholesterol staining using Filipin III was performed as per manufacturer's instructions. All wells were scanned and images collected using the InCell 2000 (GE). Image analysis was performed using ImageJ (http://imagej.nih.gov/ij/). All images were treated as stacks and adjusted for brightness, background and threshold using the same parameters. Data on signal intensity and signal area were collected using the function “Analyse Particles.” Three independent cell preparations were analyzed in triplicate for each condition.

### Statistical tests

All statistical analyses were performed in GraphPad Prism v6.05 (Graphpad Software Inc.). qPCR data, phagocytosis and cholesterol assay data was analyzed using an independent samples *t*-test whilst lysosomal enzyme activity was analyzed using One-way ANOVA with Tukey *post-hoc* test.

## Results

### Gene expression profiling of symptomatic and late-stage astrocytes

The transcriptomics study performed here is the first to complete an analysis of astrocytes from multiple stages of disease in the SOD1^G93A^ mouse model of ALS. There is an expected up-regulation of many genes involved in astrocyte activation [e.g., glial fibrillary acidic protein (*Gfap*; 90d: ~+3, 120d: +10.48), *Cd44* (90d: +2.72; 120d: +5.56)] and inflammation signaling a reactive astrocyte phenotype. In support of this, a profound up-regulation of lysosomal genes is observed, which may reflect a general increase in astrocyte reactivity already seen in ALS and could be a response to increased phagocytic activity of SOD1^G93A^ astrocytes. Concurrently, SOD1^G93A^ astrocytes continue to decrease the expression of transcripts involved in the support of neurons such as those involved in maintaining homeostasis and neuronal support; steroid and cholesterol biosynthesis, neurotransmitter receptors and potassium and sodium ion channels important for sensing alterations in the synaptic space.

All GeneChips from the symptomatic and late-stage time-points were similar in terms of percentage present call (mean = 50.42% range = 36.27–57.81%) and average background signal (mean = 33.41 range = 30.47–35.74) and GeneChips within each time-point fell within the scale factor of three recommended by Affymetrix. At the symptomatic time-point 276 transcripts (corresponding to 321 probe sets due to multiple probe sets per transcript) were differentially expressed with fold change ≥2 and *p* < 0.05 in SOD1^G93A^ astrocytes compared to NTg controls with 117 transcripts (139 probe sets) showing decreased expression and 159 transcripts (182 probe sets) increased expression. At late-stage 1685 transcripts (2066 probe sets) were differentially expressed with fold change ≥2 and *p* ≤ 0.05, with 1330 transcripts (1595 probe sets) showing decreased expression and 355 transcripts (471 probe sets) with increased expression. A list of genes with FC ≥ 2 and *p* < 0.05 for both symptomatic and late-stage categorized by gene function is shown in Supplementary Datasheet 1 [CEL files for each of the 12 GeneChips have been uploaded to the Gene Expression Omnibus Repository (GSE69166)]. PCR performed on glial cell markers before and after picking showed the astrocyte marker GFAP to still be present in LCM material, whilst levels of different cellular markers in array material shows astrocytes to be a major component of the picked material (Supplementary Figure 1). Therefore, the RNA analyzed here is from a mixed cell population with a significant astrocytic component.

Enrichment analysis was performed for genes that were differentially expressed at symptomatic and late-stage time-points to assess genes involved from disease onset (Table 1 and Supplementary Datasheets 1–6). This showed symptomatic and late-stage SOD1^G93A^ astrocytes to be very similar in terms of gene expression. The most enriched categories for up-regulated transcripts by DAVID enrichment score consisted of the lysosome, positive regulation of immune response and positive regulation of phagocytosis, pointing to an increased reactivity of astrocytes beginning at symptom onset. Potassium transport was down-regulated at the symptomatic stage which developed into a more general disruption in ion homeostasis at late-stage, which also featured dysregulation of ATP biosynthesis genes. This may signify an increased inability of astrocytes from symptomatic to late-stage disease to sense their environment.

**Table 1:** **Enrichment analysis of transcripts differentially expressed in both the (A) symptomatic and (B) late-stage time-points in SOD1^G93A^ astrocytes vs. NTg controls (***p*** ≤ 0.05, FC ≥ 2)**.

	**Term**	**GO**	**Count**	**%**	**Enrichment score**
**A**					
**UP-REGULATED TRANSCRIPTS**					
1	Positive regulation of phagocytosis	0050766	7	4.46	5.94
2	Lysosome	0005764	11	7	5.22
3	Phagocytosis	0006909	8	5.1	4.01
4	Regulation of B cell mediated immunity	0002712	6	3.82	4
5	IgG binding	0019864	4	2.55	3.32
6	T cell proliferation	0042098	5	3.18	2.85
7	Positive regulation of B-cell mediated immunity	0002714	4	2.55	2.74
8	Regulation of type III hypersensitivity	0001803	3	1.91	2.55
9	Regulation of actin cytoskeleton organization	0032956	5	3.18	2.39
10	Positive regulation of apoptosis	0043065	8	5.10	2.08
**DOWN-REGULATED TRANSCRIPTS**					
1	Voltage-gated cation channel activity	0022843	8	6.84	5.08
2	Cation channel activity	0005261	9	7.69	4.47
3	Ion channel activity	0005216	10	8.55	4.44
4	Potassium ion binding	0030955	7	5.98	3.67
5	Cellular ion homeostasis	0006873	7	5.98	1.95
6	Regulation of neuron differentiation	0045664	4	3.42	1.43
7	Cellular metal ion homeostasis	0006875	4	3.42	1.4
8	Positive regulation of immune response	0050778	4	3.42	1.18
9	Metal ion binding	0046872	23	19.66	0.92
10	Skeletal muscle tissue development	0007519	3	2.56	0.87
**B**					
**UP-REGULATED TRANSCRIPTS**					
1	Lysosome	0005764	16	4.64	4.55
2	Complement activation, classical pathway	0006958	6	1.74	3.33
3	Positive regulation of phagocytosis	0050766	6	1.74	2.99
4	Positive regulation of cell migration	0030335	6	1.74	2.57
5	Positive regulation of immunoglobulin mediated immune response	0002891	4	1.16	2.30
6	Negative regulation of protein kinase activity	0006469	6	1.74	2.07
7	Protein maturation by peptide bond cleavage	0051605	7	2.03	2.02
8	Positive regulation of type II hypersensitivity	0002894	3	0.87	2.01
9	Regulation of phosphorylation	0042325	15	4.35	1.91
10	Positive regulation of adaptive immune response	0002821	5	1.45	1.90
**DOWN-REGULATED TRANSCRIPTS**					
1	Ion channel activity	0005216	58	4.36	9.14
2	Adenyl nucleotide binding	0030554	160	12.02	8.09
3	Modification-dependent macromolecule catabolic process	0043632	66	4.96	5.81
4	Nucleotide biosynthetic process	0009165	28	2.1	4.15
5	Hydrolase activity, acting on acid anhydrides, catalyzing transmembrane movement of substances	0016820	17	1.28	3.19
6	ATP metabolic process	0046034	16	1.2	2.56
7	ATP biosynthetic process	0006754	15	1.13	2.46
8	Chloride channel activity	0005254	11	0.83	1.88
9	Neurofilament cytoskeleton	0060053	5	0.38	1.71
10	Cyclase activity	0009975	6	0.45	1.66

*Category names have been summarized from the most significant cluster annotation terms. GO, gene ontology term code; Count, number of genes; %, proportion of total gene list; Enrichment score, DAVID enrichment score (higher values are more significant)*.

### Lysosomal up-regulation occurs at symptomatic stage and continues to late-stage disease

**Figure 1:**
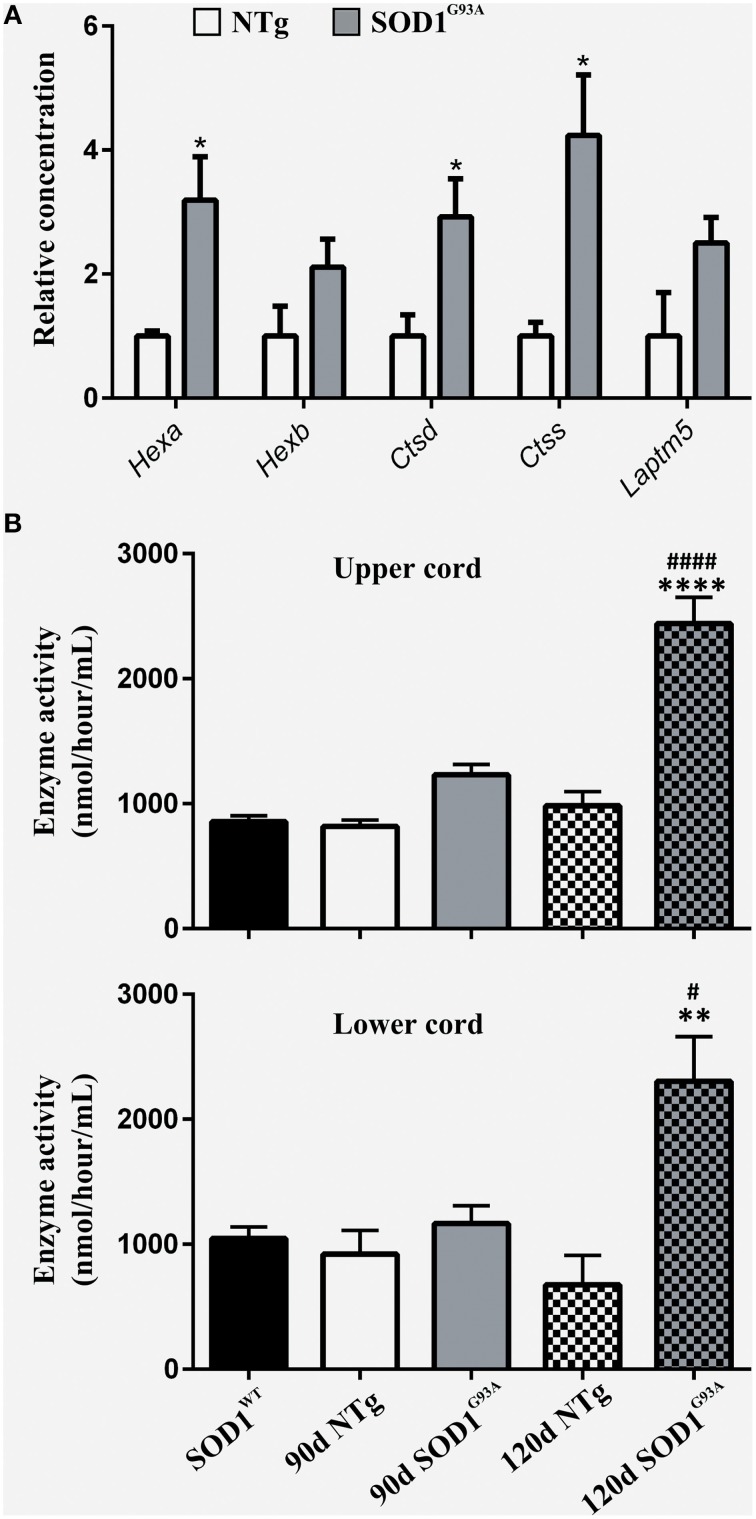
**(A)** qPCR upon symptomatic RNA of five lysosomal transcripts differentially expressed on symptomatic and late-stage microarrays; *n* = 3 per group. **(B)** Enzyme activity of Hexosaminidase B in the upper and lower spinal cord of SOD1^WT^, SOD1^G93A^, and NTg controls (SOD1^WT^, 90d NTg, and 90d SOD1^G93A^: *n* = 3, 120d NTg and 120d SOD1^G93A^: *n* = 4). Error bars = S.E.M. ^*^p ≤ 0.05, ^**^p ≤ 0.01, ^****^p ≤ 0.0001 vs. age-matched NTg control. ^#^*p* ≤ 0.05, ^*####*^*p* ≤ 0.0001 vs. SOD1^WT^ control.

To functionally validate the findings of the gene expression data, we chose to investigate the increased reactivity of astrocytes that is apparent at the symptomatic stage and continues to late-stage disease. Due to the enrichment for lysosomal genes at both time-points, we chose to investigate this group of genes first. There is a clear up-regulation of lysosomal genes in SOD1^G93A^ astrocytes at both symptomatic and late-stage time-points (Table 2). The genes involved include several lysosomal digestive proteases: cathepsin D (*Ctsd*), cathepsin H (*Ctsh*), cathepsin S (*Ctss*), cathepsin Z (*CtsZ*); two glycosidase subunits: hexosamindase A (*Hexa*) and hexosaminidase B (*Hexb*); and two minor lysosomal membrane proteins: solute carrier family 11 member 1 (*Slc11a1*) and lysosomal associated transmembrane protein 5 (*Laptm5*). Out of five genes chosen for validation by qPCR, all showed regulation in the same direction as the microarrays and three genes (*Hexa, Ctsd, and Ctss*) showed a statistically significant difference between disease and control (Figure 1A). This indicated that several aspects of lysosomal activity are increased in the SOD1^G93A^ spinal cord as the disease progresses. To assess whether the differential expression of these genes translates to a functional difference in lysosomal enzyme activity we assayed the activity of β-hexosaminidase (β-hex) in the upper and lower spinal cord. β-hex is encoded by the *Hexa* and *Hexb* transcripts (Mahuran, 1995). B-hex activity was significantly increased compared to controls in the upper cord and lower cord at the late-stage of disease (Figure 1B). As in our previous work upon motor neurons from different disease stages of the SOD1^G93A^ mouse, no differences were observed between NTg littermates and mice overexpressing wild-type human SOD1 and NTg littermates were used for all further experiments to reduce the number of animals used.

**Table 2:** **Lysosomal genes differentially expressed with ***p*** ≤ 0.05 and fold-change ≥2 in SOD1^G93A^ astrocytes vs. NTg controls**.

**Gene**	**Full name**	**Affymetrix probeset ID**	**Symptomatic FC**	**Late-stage FC**
*CD164*	cd164	1416440_at		−3.2
*CD68*	cd68	1449164_at	+4.16	+3.74
*Ctsb*	Cathepsin B	1444987_at		+2.34
*Ctsd*	Cathepsin D	1448118_a_at	+2.32	+3.28
*Ctsh*	Cathepsin H	1418365_at	+2.4	+2.77
		1443814_x_at	+2.17	+3.25
*Ctss*	Cathepsin S	1448591_at	+2.53	+4.07
*Ctsz*	Cathepsin Z	1417868_a_at	+3.22	+3.62
		1417869_s_at	+2.91	+4.67
		1417870_x_at	+3.12	+3.21
*Hexa*	Hexosaminidase A	1449024_a_at	+2.26	+2.38
*Hexb*	Hexosaminidase B	1460180_at	+2.54	+2.96
*Hgsnat*	Heparan-alpha-glucosaminide N-acetyltransferase	1436580_at		+2.18
*Laptm4b*	Lysosomal-associated protein transmembrane 4B	1416148_at		−2.92
*Laptm5*	Lysosomal-associated transmembrane protein 5	1426025_s_at	+2.52	+4.5
		1459841_x_at	+2.81	+6.45
		1417721_s_at		+3.13
		1436905_x_at		+6.45
*Slc15a3*	Solute carrier family 15, member 3	1420697_at	+3.18	+2.79

### Enhanced reactivity of SOD1^G93A^ astrocytes is demonstrated by increased phagocytic activity

**Figure 2:**
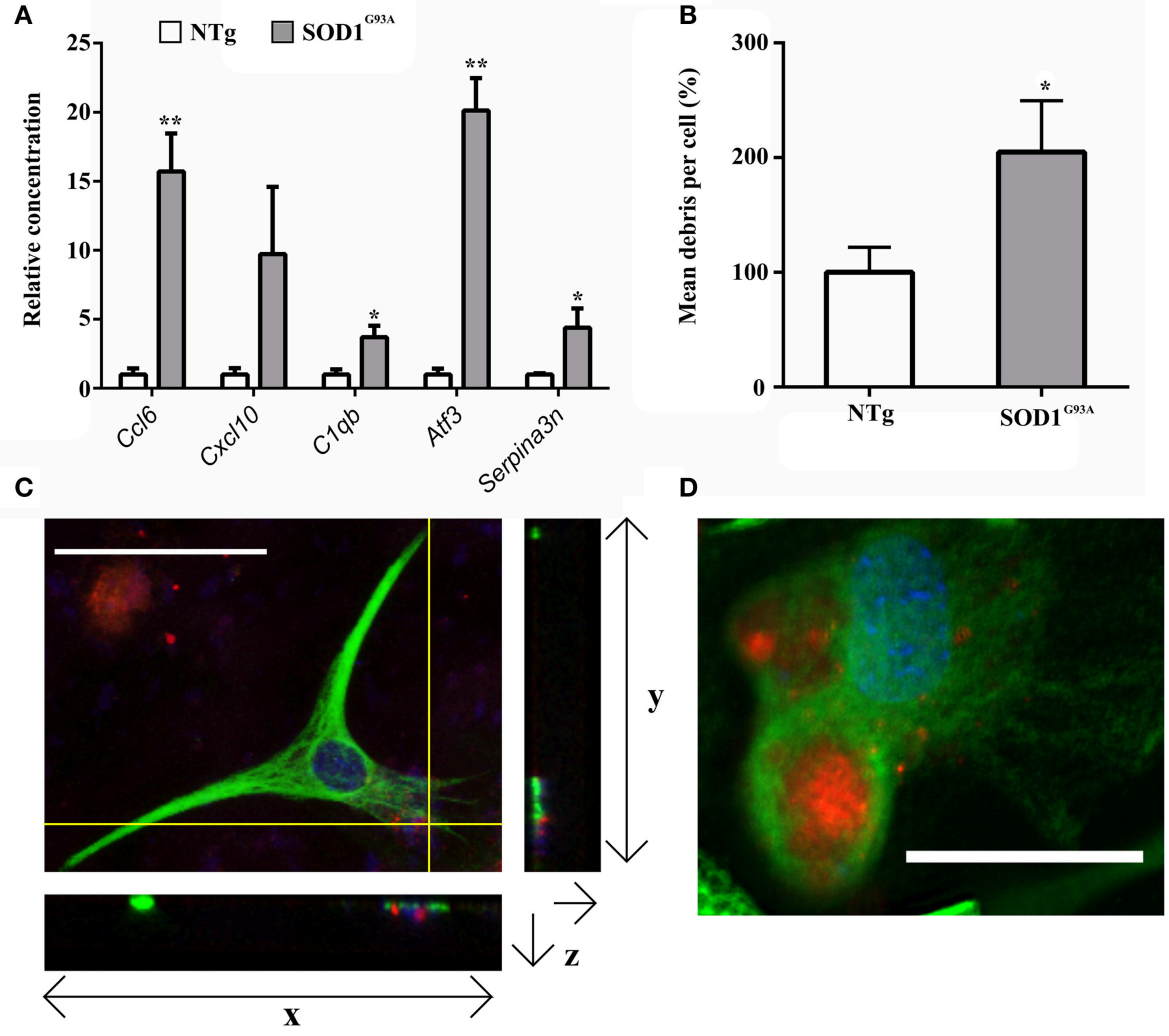
**(A)** qPCR upon symptomatic RNA of five transcripts differentially expressed in symptomatic and late-stage SOD1^G93A^ astrocyte microarray data and putatively involved in their immuno-reactive phenotype, including the chemokines *Ccl6* and *Cxcl10*, the complement component *C1qb*, the stress response transcription factor *Atf3* and the serpin peptidase inhibitor *Serpina3n*; *n* = 3 per group. **(B)** SOD1^G93A^ astrocytes phagocytose a significantly greater area of debris vs. NTg control. Results expressed as a percentage of control (NTg = 100% ± S.E.M. 12.62, SOD1^G93A^ = 204.84% ± S.E.M = 25.88). *n* = 3. **(C)** Confocal microscopy reveals NSC34 debris (red) to be in the same plane as GFAP staining (green). Crosshairs indicate location of zy and zx view. Note colocalisation of debris and GFAP in zx field. Both nuclei of astrocytes and nuclear debris of NSC34 cells are observed stained blue. **(D)** InCell image of GFAP^+^ astrocytes (green) phagocytosing NSC34 cell debris (red), nuclei are stained in blue. ^*^p ≤ 0.05, ^**^p ≤ 0.01. Error bars = S.E.M. Scale bars = 25 μm.

Gene categories relating to the immune response are up-regulated at both the symptomatic and late-stage time-points in SOD1^G93A^ astrocytes (Table 3). There is an up-regulation of chemotactic genes, with seven genes in the KEGG chemokine signaling pathway differentially expressed in symptomatic SOD1^G93A^ astrocytes (Table 3). Only two of these genes, *Ccl6* and *Cxcl10* are differentially expressed at late-stage. *Ccl6* but not *Cxcl10* was validated by qPCR (Figure 2A). The complement pathway, an innate immune mechanism for the destruction of invading pathogens, is up-regulated at both disease stages, with *C1qb* validated by qPCR as differentially expressed (Figure 2A). The complement pathway consists of a cascade of protein activation, centered around C1q, the three components of which (C1qa, b, and c) are up-regulated at symptomatic and further at late-stages in our microarray of astrocytes with C1q and C4b showing the most highly significant differential expression (*p* ≤ 0.0001) of all dysregulated genes at late stage disease. C1q binds to antigen-antibody and stimulates phagocytic activity (van Beek et al., 2003) and several other stimulants of phagocytosis are also up-regulated such as *Clec7a* and *Ptx3*. Along with these classic immune response transcripts, markers of astrocyte reactivity were also up-regulated such as activating transcription factor 3 (*Atf3*) and serpin peptidase inhibitor clade A member 3 (*Serpina3n*), which were both confirmed as differentially expressed by qPCR (Figure 2A). We next investigated whether the increased immuno-reactive phenotype of astrocytes led to differences in phagocytic ability. Cortical astrocytes from neonatal mice were used due to the difficulty of obtaining a sufficient number of proliferating astrocytes from adult spinal cord (data not shown). Astrocytes expressing SOD1^G93A^ phagocytosed significantly higher amounts of debris compared with NTg controls [204.84% (SOD1^G93A^) vs. 100% (NTg), S.E.M = 25.88 (SOD1^G93A^) vs. 12.62 (NTg), *p* = 0.0112] as measured using the InCell 2000 (Figure 2B). The InCell system was used for phagocytosis analysis due to the ability to run the assay in a 384-well format and allow automated image analysis. In order to check that debris had been engulfed and were not simply stuck to the cell surface of astrocytes, confocal microscopy was then used to confirm that debris were localized in the same plane as the astrocyte marker GFAP (Figure 2C) adding to the observation of prominent GFAP staining seen around engulfed material during InCell analysis (Figure 2D). In addition, the use of a phagocytic inhibitor, Latrunculin, abolished phagocytosis of NSC34 cell debris (Supplementary Figure 2).

**Table 3:** **Genes involved in the phagocytic and immune response categories that are differentially expressed in symptomatic and late-stage SOD1^G93A^ astrocytes vs. NTg controls (*p* ≤ 0.05, FC ≥ 2)**.

**Gene**	**Full name**	**Affymetrix probeset ID**	**Symptomatic FC**	**Late-stage FC**
**REGULATION OF PHAGOCYTOSIS**				
*fcgr1*	Fc receptor, IgG, high affinity I	1417876_at	+2.56	
*Fcer1g*	Fc receptor, IgE, high affinity I, gamma polypeptide	1418340_at	+2.58	+3.00
*Slc11a1*	Solute carrier family 11 (proton-coupled divalent metal ion transporters), member 1	1420361_at	+2.87	+3.20
*Clec7a*	C-type lectin domain family 7, member a	1420699_at	+16.26	+11.38
*Ptx3*	Pentraxin related gene	1418666_at	+2.04	+2.83
*Fcgr2b*	Fc receptor, IgG, low affinity IIb	1435477_s_at	+2.35	
*Fcgr3*	Fc receptor, IgG, low affinity III	1448620_at	+2.85	+4.00
**COMPLEMENT CASCADE**				
*Itgb2*	Integrin beta 2	1450678_at	+2.85	
*A2m*	Alpha-2-macroglobulin	1434719_at	+3.28	+11.38
*C1qa*	Complement component 1	1417381_at	+3.97	+7.98
*C1qb*	Complement component 1	1417063_at	+3.4	+4.21
		1434366_x_at	+2.36	+8.6
		1437726_x_at	+2.25	+8.89
*C1qc*	Complement component 1	1449401_at	+3.91	+5.75
*C3*	Complement component 3	1423954_at		+9.44
*C3ar1*	Complement component 3a receptor 1	1442082_at	+2.37	
*C4b*	Complement component 4b	1418021_at	+6.27	+28.67
*Cd55*	cd55	1418762_at	−2.48	
		1443906_at	−2.04	
**CHEMOKINE SIGNALING**				
*Adcy7*	Adenylate cylase 7	1450065_at	+3.02	
*Ccl3*	Chemokine (C-C motif) ligand 3	1419561_at	+9.01	
*Ccl4*	Chemokine (C-C motif) ligand 4	1421578_at	+2.12	
*Ccl6*	Chemokine (C-C motif) ligand 6	1417266_at	+4.42	+2.85
*Ccl9*	Chemokine (C-C motif) ligand 9	1417936_at	+2.05	
*Cxcl10*	Chemokine (C-X-C motif) ligand 10	1418930_at	+3.54	+10.37
*Dock2*	Dedicator of cyto-kinesis 2	1422808_s_at	+2.54	
**OTHER/REACTIVITY**				
*Atf3*	Activating transcription factor 3	1449363_at	+6.82	+4.23
*Serpina3n*	Serine peptidase inhibitor, clade A, member 3n	1419100_at	+3.90	+8

### Cholesterol processing transcripts are dysregulated in late-stage SOD1^G93A^ astrocytes

**Figure 3:**
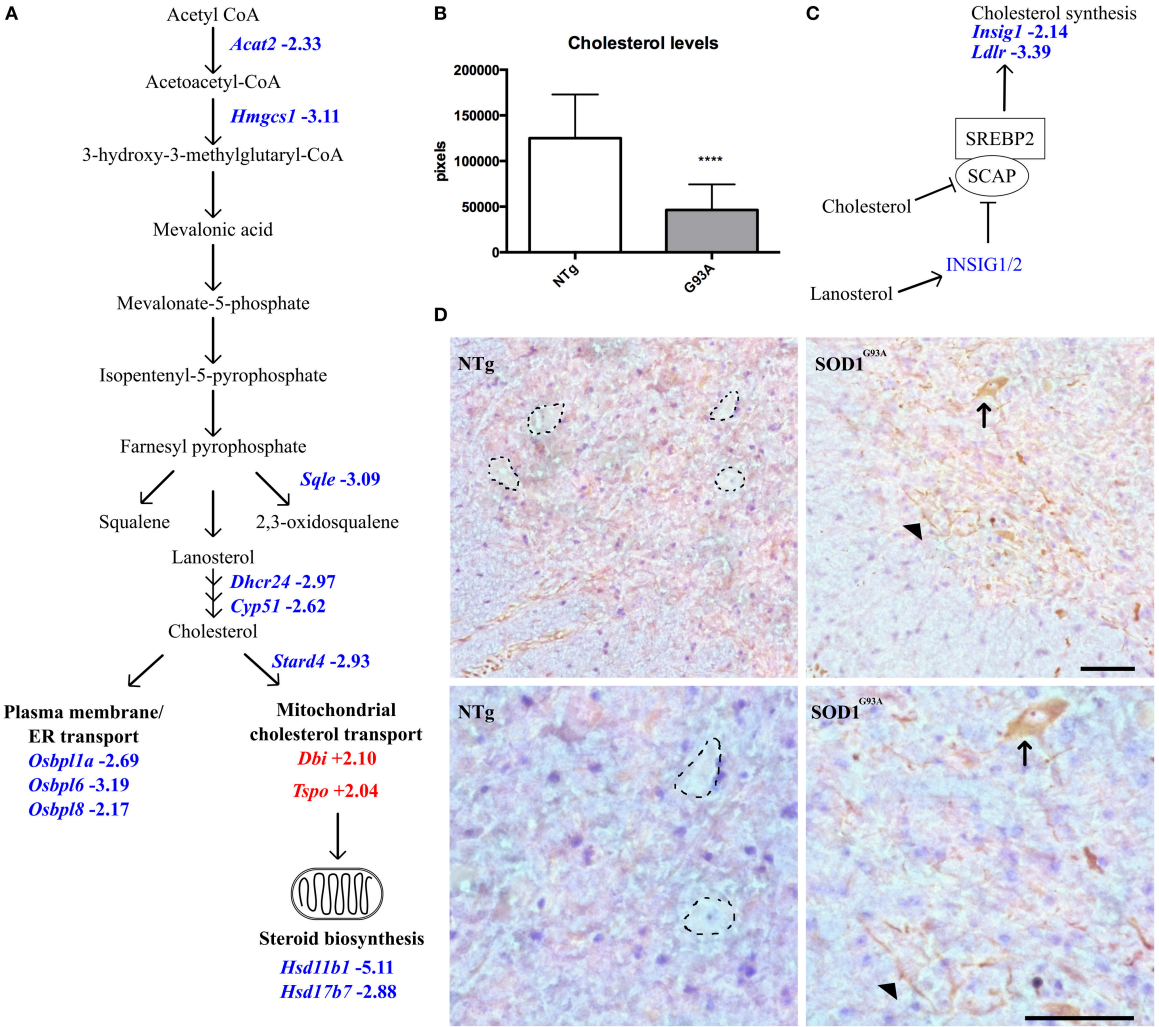
**(A)** Transcripts involved in intracellular cholesterol synthesis and transport are dysregulated in late-stage SOD1^G93A^ astrocytes vs. NTg controls. Blue indicates decreased expression, red indicates increased expression, number indicates fold-change vs. NTg control. **(B)** Intracellular cholesterol levels in neonatal SOD1^G93A^ astrocytes are significantly lower than those in NTg controls. **(C)** Simplified schematic of SREBP2 regulation. Excess cholesterol or lanosterol inhibits SCAP-mediated transport of SREBP2 to the golgi apparatus. Consequently, SREBP2 is not activated and does not translocate to the nucleus to upregulate cholesterol synthesis and genes such as *Insig1* and *Ldlr* which are decreased in late-stage SOD1^G93A^ astrocytes. **(D)** SREBP2 staining in the ventral horn of NTg (left) and SOD1^G93A^ (right) lumbar spinal cord of late-stage mice, top panels indicate 20X magnification and bottom panels 40X magnification. In NTg spinal cord, negatively stained areas show the morphology of motor neurons (dotted lines) whereas in SOD1^G93A^ spinal cord cells with motor neuron morphology stain positively for SREBP2 in the cytoplasm (arrow). In addition SREBP2 appears to be located in long processes which may belong to astrocytes or neurons (arrowhead). Scale bars = 100 μm. ^****^p ≤ 0.0001.

Whilst analysing the symptomatic and late-stage datasets, we noticed that there were a large number of differentially expressed transcripts involved in cholesterol synthesis in late-stage SOD1^G93A^ astrocytes that had not been identified by DAVID analysis (Table 4). When the late-stage gene list is analyzed in isolation this category contains 38 transcripts passing a fold-change ≥2, the majority of which (35 transcripts) are down-regulated, with several genes representing key components of the cholesterol synthesis pathway (Figure 3A). Measurement of intracellular cholesterol levels in neonatal SOD1^G93A^ astrocytes after inhibiting cholesterol export showed that transgenic astrocytes produce less than half the cholesterol of NTg control astrocytes (Figure 3B). To investigate whether cholesterol synthesis was affected in SOD1^G93A^ spinal cord, we performed staining for sterol regulatory binding protein 2 (SREBP2), a master regulator of cholesterol synthesis genes. SREBP2 is a transcription factor which is processed and re-located to the nucleus during low cholesterol conditions to up-regulate cholesterol synthesis genes (Figure 3C). SREBP2 is also involved in the transcription of the low density lipoprotein receptor (LDLR) which is down-regulated at the transcript level (-3.39) in late-stage SOD1^G93A^ astrocytes. *Srebp2* is not down-regulated at the transcript level in either symptomatic or late-stage astrocytes. However, in late-stage SOD1^G93A^ spinal cord we saw an increased staining for SREBP2 in cells with motor neuron morphology and within processes that may belong to astrocytes (arrowhead in Figure 3D). In contrast NTg spinal cord contains no such astrocytic processes and negatively stained areas with motor neuron morphology. Taken together, the increased staining of SREBP2 in the cytoplasm of SOD1^G93A^ motor neurons may be an attempt by neurons to increase cholesterol synthesis because of reduced astrocytic cholesterol provision.

**Table 4:** **Transcripts involved in cholesterol homeostasis that are differentially expressed in symptomatic and late-stage SOD1^G93A^ astrocytes vs. NTg controls (***p*** ≤ 0.05, FC ≥ 2)**.

**Gene**	**Full name**	**Affymetrix probeset ID**	**Symptomatic FC**	**Late-stage FC**
*Abca5*	ATP-binding cassette, sub-family A (ABC1), member 5	1434474_at		−3.21
*Abcb7*	ATP-binding cassette, sub-family B (MDR/TAP), member 7	1435006_s_at		−2.44
*Abcc8*	ATP-binding cassette, sub-family C (CFTR/MRP), member 8	1455765_a_at		−2.06
*Abcd2*	ATP-binding cassette, sub-family D (ALD), member 2	1456812_at		−2.58
*Acat2*	Acetyl-Coenzyme A acetyltransferase 2	1435630_s_at		−2.33
*Acsl4*	Acyl-CoA synthetase long-chain family member 4	1433531_at		−2.35
*Acsl6*	Acyl-CoA synthetase long-chain family member 6	1437031_at		−2.59
*Acss2*	Acyl-CoA synthetase short-chain family member 2	1422478_a_at		−2.23
*ApoE*	Apolipoprotein E	1432466_a_at	+2.18	+5.23
*Capn7*	calpain 7	1423096_at		−2.00
*Ch25h*	Cholesterol 25-hydroxylase	1449227_at		−2.63
*Cyp51*	Cytochrome P450, family 51	1450646_at		−2.62
		1422533_at		−2.34
*Dbi*	Diazepam binding inhibitor	1433991_x_at		+2.10
		1455976_x_at		+2.04
*Dhcr24*	24-dehydrocholesterol reductase	1418129_at		−2.94
		1451895_a_at		−2.97
*Extl2*	Exostoses (multiple)-like 2	1422538_at		−3.38
		1422539_at		−2.39
*Fads6*	Fatty acid desaturase domain family, member 6	1443904_at		−2.14
*Far1*	Fatty acyl CoA reductase 1	1435315_s_at		−2.41
*Hmgcs1*	3-hydroxy-3-methylglutaryl-Coenzyme A synthase 1	1433444_at		−3.4
		1433445_x_at		−3.11
		1433446_at		−2.45
*Hsd11b1*	Hydroxysteroid 11-beta dehydrogenase 1,	1449038_at	−2.32	−5.05
*Hsd17b7*	Hydroxysteroid (17-beta) dehydrogenase 7	1457248_x_at	−2.05	−2.88
*Insig1*	Insulin induced gene 1	1454671_at		−2.14
*Insig2*	Insulin induced gene 2	1417980_a_at		−2.58
		1417981_at		−2.58
*Ldlr*	Low density lipoprotein receptor	1421821_at		−3.39
*lpl*	Lipoprotein lipase	1431056_a_at	+5.99	−11.21
*Lrp11*	Low density lipoprotein receptor-related protein 11	1433536_at		−3.96
*Lrp12*	Low density lipoprotein-related protein 12	1433864_at		−2.28
*Lypd6b*	LY6/PLAUR domain containing 6B	1429274_at		−2.84
*Nsdhl*	NAD(P) dependent steroid dehydrogenase-like	1416222_at		−2.96
*Nus1*	Nuclear undecaprenyl pyrophosphate synthase 1 homolog (S. cerevisiae)	1419914_s_at		−2.01
*Osbpl1a*	Oxysterol binding protein-like 1A	1460192_at		−2.69
*Osbpl6*	Oxysterol binding protein-like 6	1457881_at		−3.19
*Osbpl8*	Oxysterol binding protein-like 8	1437069_at		−2.17
*Sc4mol*	Sterol-C4-methyl oxidase-like	1423078_a_at		−2.97
*Sc5d*	Sterol-C5-desaturase (fungal ERG3, delta-5-desaturase) homolog (S. cerevisae)	1434520_at		−3.33
*Scd1*	Stearoyl-Coenzyme A desaturase 1	1415964_at		−2.43
*Sqle*	Squalene epoxidase	1415993_at		−3.09
*Stard4*	StAR-related lipid transfer (START) domain containing 4	1429240_at		−2.93
		1455011_at		
*Tspo*	Translocator protein	1438948_x_at		+2.04

### Overlapping gene expression of pre-symptomatic, symptomatic, and late-stage SOD1^G93A^ astrocytes

**Figure 4:**
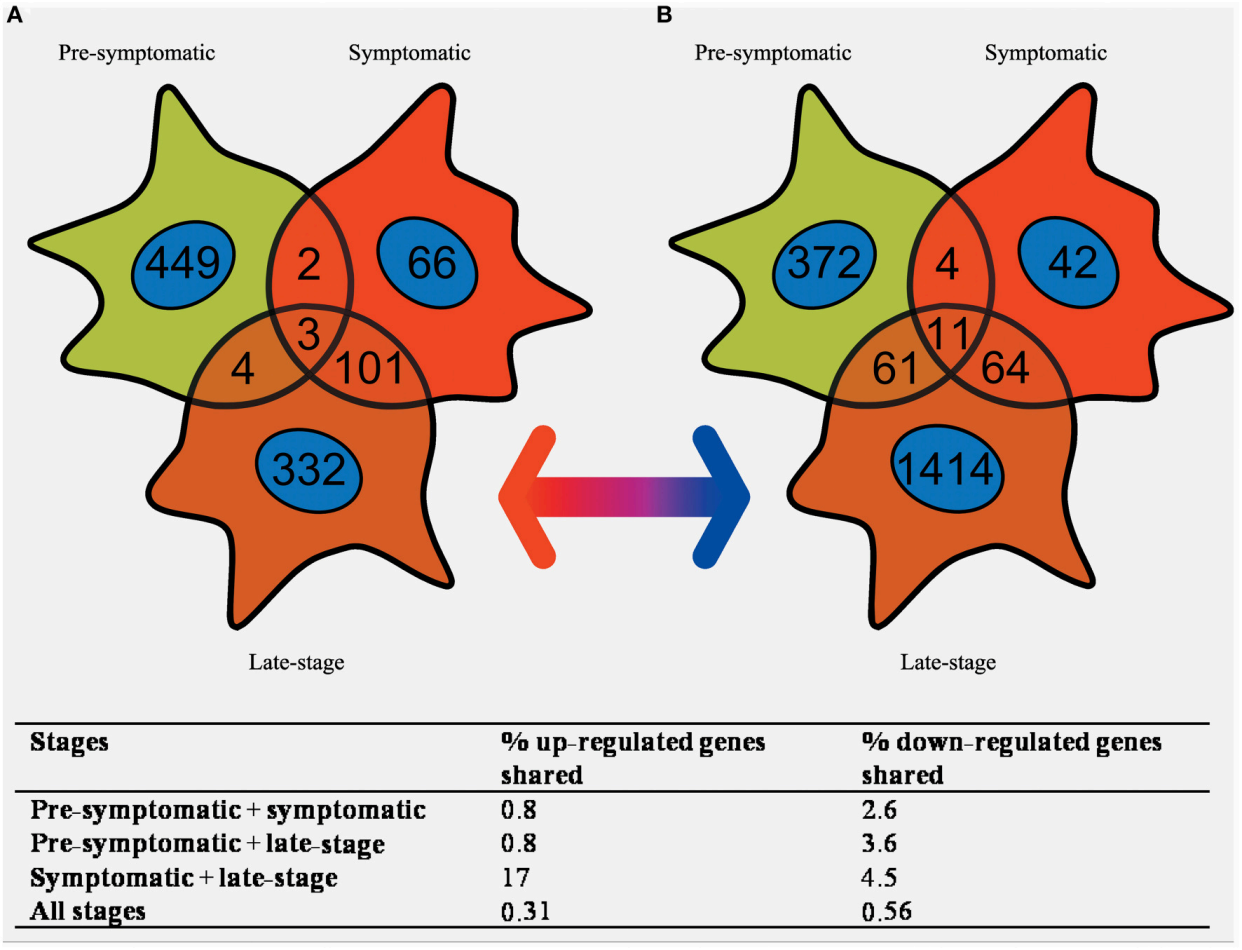
**Venn diagram of the (A) up-regulated and (B) down-regulated genes that are differentially expressed in SOD1^G93A^ astrocytes at pre-symptomatic, symptomatic, and late-stage disease**. The greatest overlap occurs between up-regulated transcripts in symptomatic and late-stage SOD1^G93A^ astrocytes. In comparison very few genes are shared between pre-symptomatic astrocytes and the other two disease stages, or between all three disease stages together.

Pre-symptomatic SOD1^G93A^ astrocytes have already undergone gene expression profiling by Ferraiuolo et al. (2011a) which enables a comparison of the gene lists generated at three time-points of the disease. The number of transcripts with differential expression passing *p* ≤ 0.05 and FC ≥ 2 in common between all three time-points was investigated using a Venn diagram (Figure 4). There is high variability in the number of transcripts differentially expressed at FC ≥ 2 between the three time-points, with the symptomatic stage showing a lower number of transcripts (276) compared to the pre-symptomatic (878) and late-stage (1685) GeneChips. The highest proportion of genes shared is between the symptomatic and late-stage time-points where 17% of up-regulated and 4.5% of down-regulated transcripts are in common (for full lists of shared genes see Supplementary Datasheet 6). The separation also shows that there is a large amount of transcriptional down-regulation at late-stage disease compared to the other time-points which are more evenly split, as previously seen in our microarray studies of the corresponding time-points of SOD1^G93A^ motor neurons (Ferraiuolo et al., 2007). The overlap between the pre-symptomatic + late-stage and symptomatic + late-stage time-points is more evenly matched for down-regulated transcripts, whereas the overlap between the pre-symptomatic and symptomatic time-points is again very small. This reinforces the findings of our enrichment analyses (Table 1) which featured many common categories between the symptomatic and late-stage time-points.

## Discussion

**Figure 5:**
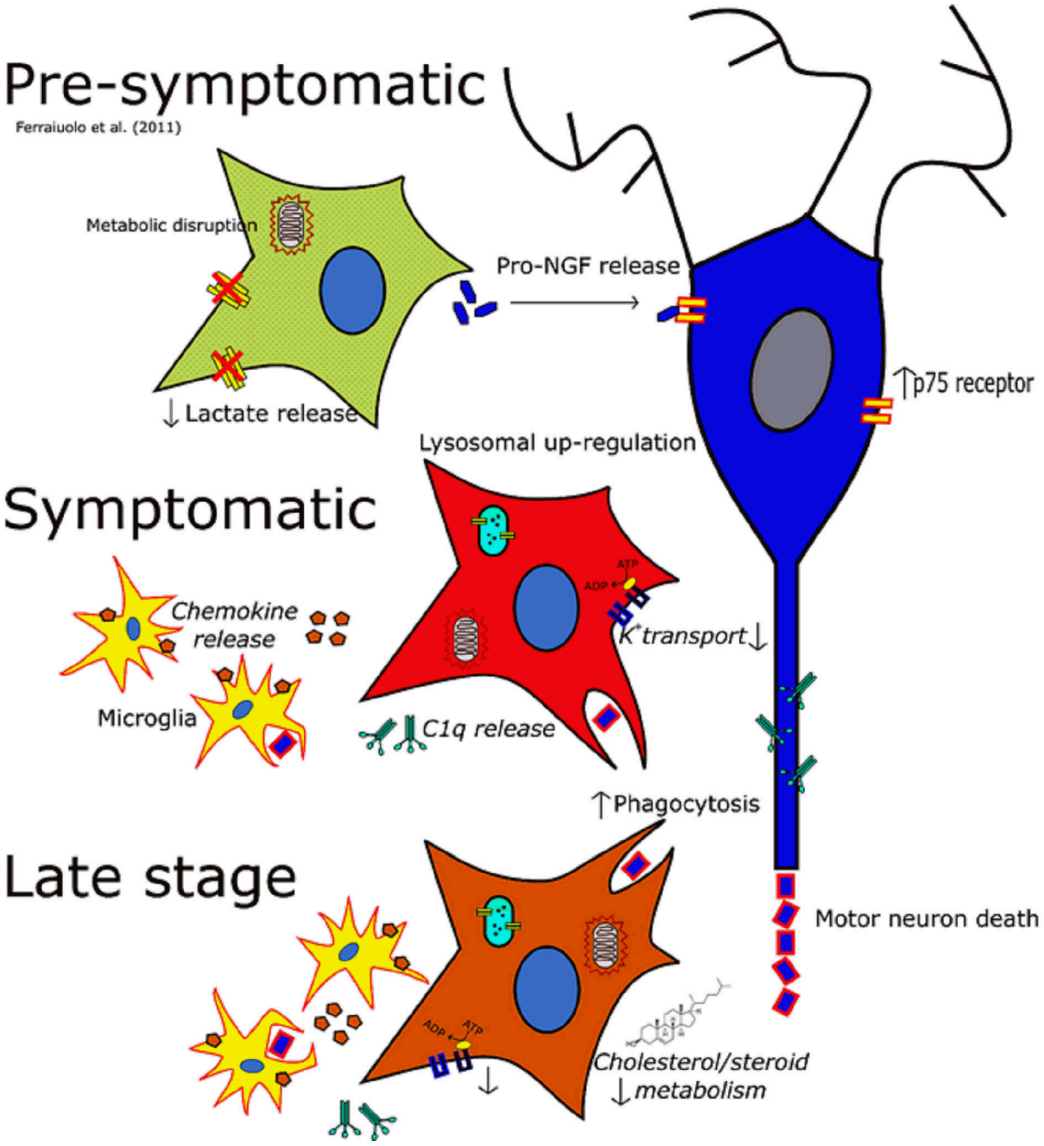
**The changing phenotype of astrocytes during the disease course in the SOD1^G93A^ mouse**. Italics indicate data inferred from microarrays whilst non-italics indicate data for which functional outcomes have been observed. At the pre-symptomatic stage astrocyte gene expression is characterized by disruptions in lactate provision to neurons and mitochondrial dysfunction, as well as perturbations in secreted trophic factors such as the pro-NGF:NGF ratio. At the symptomatic time-point astrocytes take on a much more reactive phenotype characterized by increased lysosomal, phagocytic and chemokine gene expression whilst also featuring a down-regulation of genes involved in potassium transport. At late-stage disease astrocytes feature differential expression of a large number of genes, and still show the disruption seen at the symptomatic stage. In addition cholesterol synthesis transcripts are differentially expressed at this time-point which may indicate an excess of cholesterol in the CNS, or a disruption in the activation of cholesterol synthesis.

Gene expression profiling of astrocytes from the SOD1^G93A^ transgenic mouse at symptomatic and late-stage disease time-points and subsequent combination with the previous analysis by Ferraiuolo et al. (2011a) at the pre-symptomatic stage has allowed a picture of astrocyte behavior throughout the ALS disease course in the murine model to be seen for the first time (Figure 5). The pre-symptomatic time-point is highly associated with an alteration of supportive function in SOD1^G93A^ astrocytes, such as the provision of lactate to motor neurons and altered secretion of pro-nerve growth factor (ProNGF). In the current study, we have identified an altered immune response beginning at symptomatic and continuing through to the late-stage of disease, shown by the large overlap of increased transcripts between these two stages (Figure 4). Whilst there is evidence of neuronal and microglial contamination in the LCM samples (Supplementary Figure 1), it is well established that astrocytes are highly activated during the late-stages of disease. In addition, our experiments, particularly those looking at the phagocytosis of neuronal debris and cholesterol synthesis, sought to confirm these as astrocytic phenomena by using pure cultures of astrocytes. The lysosomal up-regulation seen at the symptomatic time-point, with further up-regulation at late-stage, would be in keeping with either: attempts to deal with intracellular inclusions, and/or increased phagocytosis of motor neuronal debris. At the late-stage time-point, defects in ion homeostasis and cholesterol and steroid metabolism are also seen. The greatly increased expression of genes involved in immune function, complement, cell signaling, and secretion pathways seen at the symptomatic time-point are less apparent at late-stage, which probably reflects the widespread dysregulation of transcripts in astrocytes at this time-point leading to a lower enrichment. It is apparent from these data that a transition occurs between the pre-symptomatic and symptomatic time-points leading to a reactive astrocyte phenotype characterized by increased chemokine secretion, lysosomal up-regulation and up-regulation of stress response factors which carries through to late-stage. This is reinforced by a recent gene expression study of SOD1^G93A^ spinal cord which reported an increase in antigen presentation transcripts from an early pre-symptomatic stage (40d) to a late-pre-symptomatic stage (80d) (de Oliveira et al., 2013). For maximum applicability of functional data to both disease stages, we chose to functionally investigate this reactive response rather than the down-regulation of categories such as ion homeostasis due to the aforementioned large overlap for up-regulated transcripts (Figure 4).

The increase in expression of cathepsin transcripts is in keeping with several previous studies which reported increased expression in glia (Fukada et al., 2007) and decreased expression in motor neurons in SOD1-ALS (Wootz et al., 2006; Cox et al., 2010; Kirby et al., 2011), suggesting up-regulation of the lysosomal pathway is astrocyte-specific. Up-regulation of lysosomal proteins could be as a result of increased amounts of autophagy of SOD1^G93A^ protein, aggregates of which are degraded by autophagy *in vitro* (Kabuta et al., 2006). Interestingly, SOD1 and CtsD are co-localized in motor neurons of sALS patients (Forsberg et al., 2010). However, our results do not show an up-regulation of the classic autophagy related genes such as *Beclin 1, Lc3-II, Vps34*, or the *Atg* group of genes (Virgin and Levine, 2009). This suggests that if autophagy is increased, it is due to altered autophagic flux rather than induction of autophagic genes.

Rather than a lysosomal up-regulation due to increased autophagy, the microarray data suggests that lysosomal up-regulation could be as a consequence of increased astrocyte phagocytosis (Table 1). Indeed, in addition to the cathepsin group of proteases, this study is the first to highlight an increased β-hexosaminidase activity in SOD1^G93A^ spinal cord at the late-stage of disease (Figure 1B). The fact that both transcripts that make up the two β-hexosaminidase isoforms are differentially expressed in the microarray data makes it highly likely that part of this increased activity comes from astrocytes. β-hexosaminidase is involved in the processing of gangliosides, which are abundant upon cell membranes, and its increased activity from the symptomatic stage onwards may be in response to increased processing of neuronal debris. Astrocytes are capable phagocytes (Chang et al., 2000) and their phagocytosis of cell debris is seen to protect neurons from contact-induced cell death *in vitro* (Lööv et al., 2012). Here we compared the phagocytic ability of SOD1^G93A^ astrocytes vs. NTg controls and have shown for the first time that SOD1^G93A^ astrocytes have an increased propensity to phagocytose cellular debris (Figure 2). Again, the results of the functional study were consistent with the up-regulation of several membrane receptors for phagocytosis (Table 2). Given the data of Lööv et al. (2012), we hypothesize that this would actually function as a protective mechanism during the disease course and dampen inflammation. In support of this, macrophage phagocytosis of either apoptotic or necrotic debris does not affect expression of pro-inflammatory cytokines (Brouckaert et al., 2004).

At the late-stage of disease in particular, many transcripts involved in cholesterol and steroid homeostasis are dysregulated in SOD1^G93A^ astrocytes. Astrocytes are important in the adult CNS as they provide cholesterol and cholesterol precursors to neurons via transport proteins such as APOE (Pfrieger and Ungerer, 2011). Another mouse model which closely resembles ALS, featuring a deletion of the hypoxia response element of the vascular endothelial growth factor gene (VEGF^δ∕δ^), shows down-regulation of many of the transcripts seen in the present study including: *Ldlr, Stard4, Hsd17b7*, and *Sqle* (Brockington et al., 2010). In contrast to the results of ourselves and Brockington et al. (2010) showing decreased cholesterol synthesis, Ceramide 24 and sphingomyelin (classes of membrane lipid) and cholesterol-16 and -18 are increased in the spinal cord of ALS patients and in the lumbar spinal cord of the pre-symptomatic SOD1^G93A^ mouse (Cutler et al., 2002). The decreased intracellular cholesterol measured in the current study may be the reason for increased translocator protein (*Tspo*) (120d: +2.04), which along with diazepam binding inhibitor (*Dbi*) (120d: +2.4) induces translocation across the mitochondrial membrane (Rone et al., 2009). This may be an attempt by the cell to increase cholesterol transport into mitochondria for steroid biosynthesis. However, downstream steroid biosynthesis within mitochondria is predicted to be decreased due to the decreased expression of *Hsd11b1* and *Hsd17b7*. This decreased expression is superimposed upon the dysregulation of mitochondrial transcripts already apparent in the pre-symptomatic SOD1^G93A^ astrocytes (Ferraiuolo et al., 2011a). Future therapeutic strategies could focus upon mitochondrial protection to rescue this deficit and thereby increase production of neuroprotective steroids.

We show here that SOD1^G93A^ astrocytes inherently produce a lower amount of cholesterol than NTg controls. This is the first time to our knowledge that this has been demonstrated. The increased APOE activity seen in the microarray data might therefore be an attempt to increase cholesterol transport in response to lower astrocytic cholesterol synthesis. Future study should focus upon confirming the increased expression of APOE at the protein level. The increased staining of SREBP2 in motor neuronal cell bodies and process-like structures of late-stage SOD1^G93A^ lumbar spinal cord may result from a neuronal reaction to lower cholesterol transport to neurons from astrocytes, and an up-regulation of SREBP2 by neurons in an attempt to synthesize cholesterol themselves. To investigate this further, *in vitro* studies should be performed to investigate whether increased extracellular cholesterol leads to cytoplasmic SREBP2 localization in astrocytes or motor neurons and whether the SOD1^G93A^ mutation affects this.

This study has revealed three previously unexplored phenomena in SOD1^G93A^ astrocytes occurring at the symptomatic stage and continuing to late-stage disease. Intriguingly, these mechanisms may be interconnected and future studies can now focus on characterizing these behaviors further. Much focus is placed upon whether astrocytes become toxic or lose supportive function during ALS progression. The data presented in this report suggest that astrocytes react so as to attempt damage limitation via clearance and processing of cellular debris for re-distribution of components such as cholesterol. In addition, the transcriptomic data of symptomatic and late-stage SOD1^G93A^ astrocytes show decreased expression of many transcripts involved in ion homeostasis and transport, suggesting that astrocytes are concurrently losing their ability to regulate the external environment. Given this, we conclude that the majority of behavior change in SOD1^G93A^ astrocytes during disease progression is a loss of supportive function rather than a toxic gain of function and that this is equally damaging to neuronal survival. It is important now to determine whether these astrocytic behaviors can be modulated and whether this is a means by which to influence disease progression.

## Author contributions

LF, DB^2^, JK, PH, and PS were involved in the conception and design of the initial work. PV, DS, and LF were involved in the acquisition of material for microarray samples. DB^1^ and LF conceived, performed and analyzed further experiments based upon the microarray data. MK and DB^1^ performed the assay of β-hexosaminidase activity. All authors were involved in the drafting of the manuscript and final approval of the version to be published.

^1^ David Baker

^2^ Daniel Blackburn

## Funding

We acknowledge grants from the European Community's Seventh Framework Programme (FP7/2007-2013) under the EuroMOTOR project, grant agreement no 259867 and the EU Joint Programme-Neurodegenerative Disease Research (JPND) projects, SOPHIA and STRENGTH, supported through the following funding agencies under the aegis of JPND—http://www.jpnd.eu/: United Kingdom, Medical Research Council to PJS and JK. PJS is an NIHR Senior Investigator. LF is funded by a EU Marie Curie Fellowship. PV was funded by a Wellcome Trust summer studentship. MK was funded by a BBSRC/Lilly (PhD CASE studentship).

### Conflict of interest statement

The authors declare that the research was conducted in the absence of any commercial or financial relationships that could be construed as a potential conflict of interest.
