# Supplementary material for: Lysosomal and phagocytic activity is increased in astrocytes during disease progression in the SOD1 G93A mouse model of amyotrophic lateral sclerosis
Source: Front Cell Neurosci. 2015 Oct 15;9:410. doi: 10.3389/fncel.2015.00410 (PMC4606544; doi:10.3389/fncel.2015.00410)
Supplement: Supplementary file 7 [file SupplementaryFiguresandTables.PDF]

Supplementary Material

Lysosomal and phagocytic activity is increased in astrocytes during disease progression in the SOD1^{G93A} mouse model of amyotrophic lateral sclerosis

David J. Baker[1], Daniel J. Blackburn[1], Marcus Keatinge[1], Dilraj Sokhi[1], Paulius Viskaitis[1], Paul R. Heath[1], Laura Ferraiuolo[1], Janine Kirby[1]* and Pamela J. Shaw[1]*

*Equal contribution

[1]Sheffield Institute for Translational Neuroscience, University of Sheffield, 385 Glossop Road, Sheffield, S10 2HQ, United Kingdom

***Correspondence:** Dr Janine Kirby, Sheffield Institute for Translational Neuroscience, University of Sheffield, 385 Glossop Road, Sheffield, S10 2HQ, United Kingdom.

Tel: 01142222247

Email: j.kirby@sheffield.ac.uk

1 Supplementary Figures

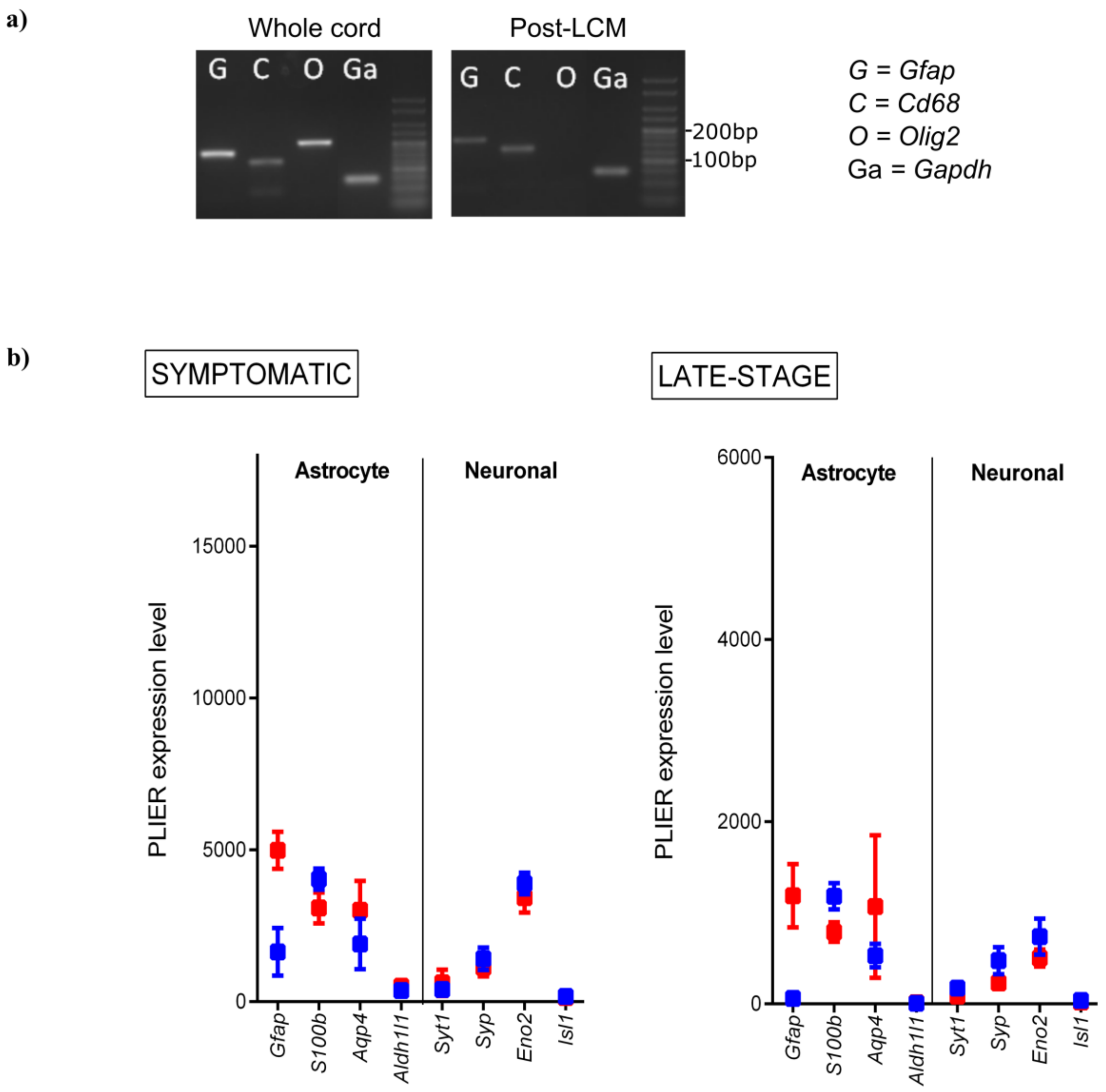

Supplementary Figure 1. Purity of RNA used for microarray analysis a) prior to LCM and after LCM and b) astrocyte and neuronal markers on microarray Genechips. LCM removed oligodendrocyte contamination however microglial and neuronal markers were present following LCM, meaning that a mixed cell population with a significant astrocyte component has been obtained. Blue = NTg samples, Red = $SOD1^{G93A}$.

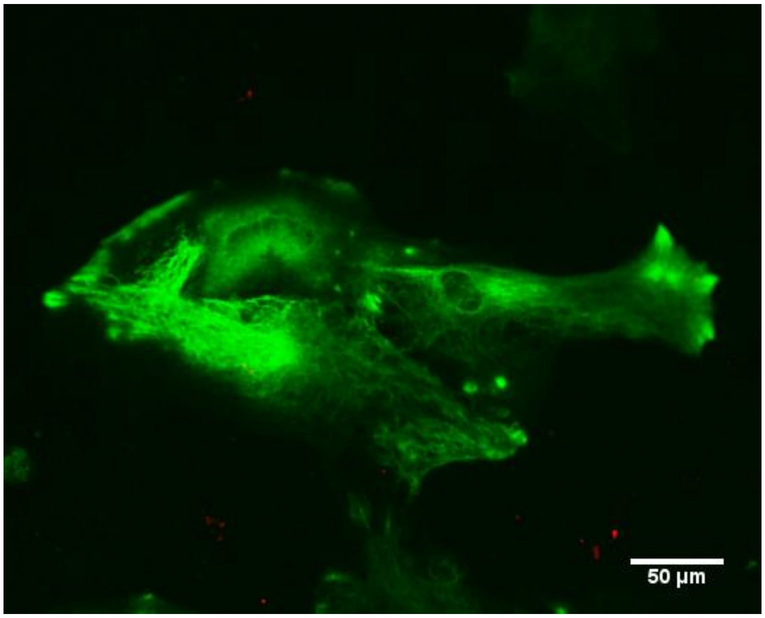

Supplementary Figure 2 Representative image of astrocytes treated with the phagocytic inhibitor Latrunculin (1µM) before being treated with NSC34 cell debris. Green = GFAP, red = cell debris, magnification = 20X.

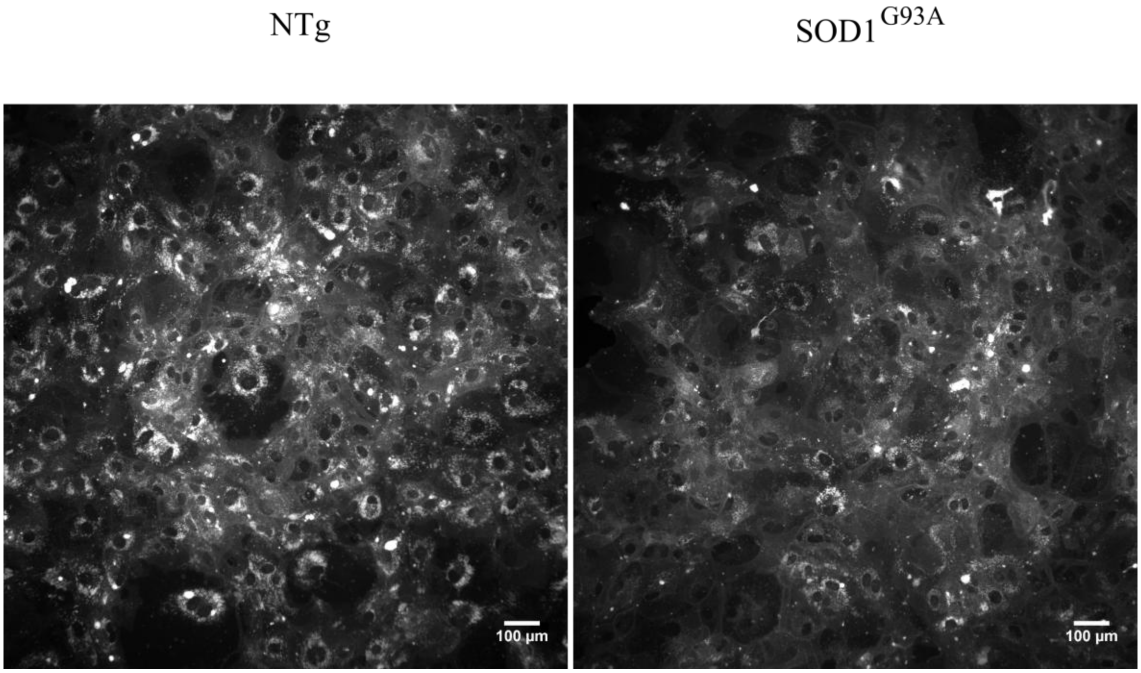

Supplementary Figure 3 Cholesterol staining using Filipin III. Staining intensity was significantly higher in NTg astrocytes versus SOD1^{G93A} ($p<0.0001$).

1.1 Supplementary Tables

Supplementary Table 1 Primer sequences and concentrations used in the qPCR validation of targets from microarray analysis of symptomatic and late-stage $SOD1^{G93A}$ astrocytes.

Category	Gene name	Gene symbol	Accession	Primer	Sequence (5'-3')	Conc. (nM)
**Lysosome**	*cathepsin D*	*Ctsd*	NM_009983.2	FW	TGCTGCCCTCTGCCTGTCTGA	150
				RV	AGGGCGGCAGGACAAGACAA	150
	cathepsin S	*Ctss*	NM_021281.2	FW	CGACGCCAGCCATTCCTCCTT	150
				RV	AGAGTCCCATAGCCAACCACAAG A	150
	hexosaminidase A (alpha polypeptide)	*Hexa*	NM_010421.4	FW	TCGCTGAGAGACTGTGGAGCAGT A	150
				RV	GGCCTGGATTCCTCTCCTCACCA	150
	hexosaminidase B (beta polypeptide)	*Hexb*	NM_010422	FW	TGCAGAATGGTCAGCCGTG	150
				RV	GAATGCTGTAGACGTCTGTCACT	150
	lysosomal protein transmembrane 5	*Laptm5*	NM_010686.3	FW	AGCACCTGGAGGCTGGGAAGTC	150
				RV	TTGCCATCAGAGCAGTGGCTCAT	150
**Chemokine**	C-X-C motif chemokine 10	*Cxcl10*	NM_021274	FW	TAGCTCAGGCTCGTCAGTTCTA	300
				RV	TGGGAAGATGGTGGTTAAGTTC	150
	chemokine (C-C motif) ligand 6	*Ccl6*	NM_009139.3	FW	GCAGGCATTGTCACCCACTT	50
				RV	AGGCGGAGTCCACATGAATAC	100
**Complement cascade**	c1qb	*C1qb*	NM_009777.2	FW	CACCAACGCGAACGAGAACT	150
				RV	GGCCAGGCACCTTGCA	150
**Stress response**	activating transcription factor 3	*Atf3*	NM_007498.3	FW	TGTACCCACTGCAGAGGAAG	100
				RV	GCCCTGTCACTGAGTATGGA	100
	serine peptidase inhibitor clade A member 3n	*Serpina3n*	NM_009252.2	FW	GCCTGGAGGATGTCCTTTCAA	300
				RV	AGCCTTGTGGACCACCTGAG	150
**Lipid metabolism**	lipoprotein lipase	*Lpl*	NM_008509.2	FW	CGGCATCCCCATTATTGCTA	300

				RV	ACTTTCCAGTGTTTACAAGCATTC	300
**House keeping**	glyceraldehyde-3-phosphate dehydrogenase	*Gapdh*	NM_008084.2	FW	GCTACACTGAGGACCAGGTTGTCT	300
				RV	AGCCCCGGCATCGAA	300
